# Cannabidiol impairs tumor-associated behaviors and modulates apoptosis- and autophagy-related pathways in cisplatin-resistant head and neck squamous cell carcinoma

**DOI:** 10.1007/s12032-026-03480-7

**Published:** 2026-10-26

**Authors:** Edilene S. A. de Melo, Joaquim M. D. Almeida, Arthur C. Pires, Rosy Iara M. A. Ribeiro

**Affiliations:** 1 Experimental Pathology Laboratory, Federal University of São João del Rei (UFSJ), Rua Sebastião Gonçalves Coelho, 400, Chanadour, Divinópolis, 35501-296 MG Brazil; 2 Plant Cell Culture Laboratory, Federal University of São João del Rei (UFSJ), Rua Sebastião Gonçalves Coelho, 400, Chanadour, Divinópolis, 35501-296 MG Brazil; 3 Laboratory of Experimental and Computational Neuroscience, Federal University of São João del Rei (UFSJ), Praça Dom Helvécio, 74 - Dom Bosco, São João del Rei - MG, 36301- 160 MG, Brazil

**Keywords:** HNSCC, Cannabidiol, Selective, Apoptosis, Autophagy

## Abstract

Head and neck squamous cell carcinoma (HNSCC) remains a challenge due to recurrence, metastasis, and resistance to conventional therapies, particularly in human papillomavirus (HPV)-negative tumors. Cannabidiol (CBD), a non-psychoactive phytocannabinoid, has demonstrated antitumor activity in several cancer models; however, its effects in chemoresistant HNSCC are not well established. This study evaluates the antitumor effects of CBD and cellular mechanisms in a cisplatin -resistant, HPV-negative HNSCC model. Cytotoxic effects of CBD were assessed in HN13 cells and compared with fibroblasts and keratinocytes using MTT and selectivity index analysis. Migration, invasion, metalloproteinase activity, clonogenic survival, and cytotoxicity on 3D spheroids were evaluated. Cell death-related mechanisms were investigated using acridine orange/propidium iodide staining and Western blot analysis of apoptosis, autophagy, and cell-cycle regulation. Antitumor activity and vascular safety were further assessed using the chorioallantoic membrane model and HET-CAM assay. The combined effect of CBD and CIS was evaluated using the combination index analysis. CBD exhibited selective cytotoxicity toward HN13 cells and reduced migration, invasion, matrix metalloproteinase-2/9 activity, clonogenic survival, and 3D spheroid growth. CBD altered proteins associated with apoptosis, autophagy, and cell-cycle regulation. Pharmacological inhibition with 3-MA enhanced CBD-associated apoptotic signaling, increasing PARP and caspase-3 cleavage. In the CAM, CBD reduced tumor growth and vascular area without inducing vascular irritation. Furthermore, CBD and CIS demonstrated synergistic cytotoxic effects in HN13 cells. CBD exerted selective antitumor effects on CIS-resistant HN13 HNSCC cells, affecting tumor-associated behaviors and modulating apoptosis and autophagy-related signaling, while acting synergistically with cisplatin. These findings support further preclinical investigation of CBD as a potential adjunct approach in HNSCC, particularly in chemoresistant settings.

**Graphical abstract:**

**Figure:**
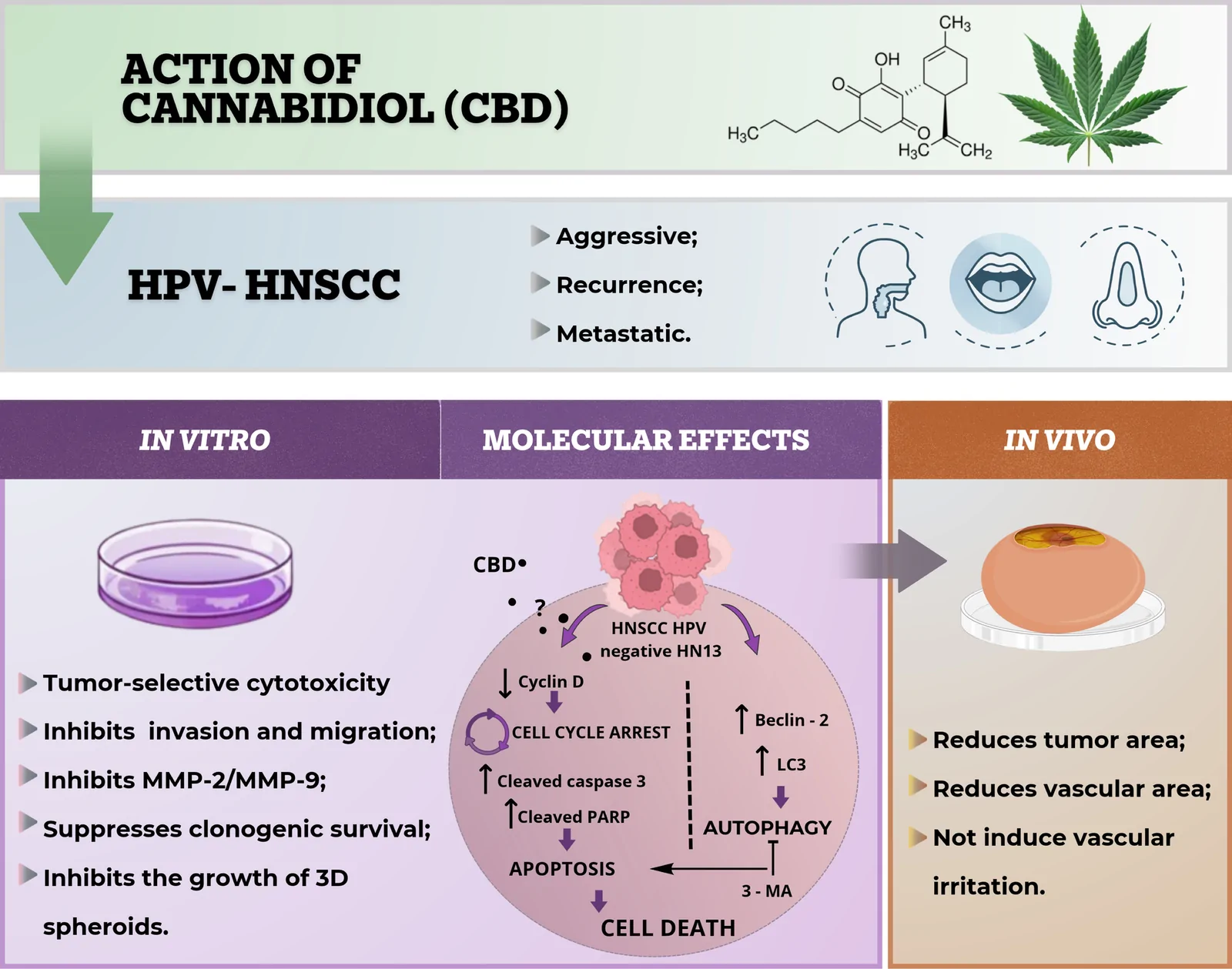

**Supplementary Information:**

The online version contains supplementary material available at https://doi.org/10.1007/s12032-026-03480-7.

## Introduction

Cancer remains a major global public health problem and a leading cause of mortality worldwide. Among solid tumors, Head and neck squamous cell carcinoma (HNSCC) is one of the most common malignancies arising from the mucosal epithelium of the oral cavity, sinuses, pharynx, larynx, and salivary glands [1]. Despite advances in surgery, radiotherapy, and systemic treatments, the overall 5-year survival rate for HNSCC remains approximately 50%, reflecting its aggressive behavior, high rates of recurrence, and strong metastatic potential [2, 3].

HNSCC comprises biologically and clinically distinct subtypes based on human papillomavirus (HPV) status. HPV-positive (HPV+) HNSCC is driven by the viral oncoproteins E6 and E7, which promote functional degradation of key cellular regulators involved in DNA repair, apoptosis, cell cycle control, and telomere maintenance, including p53, Bax, pRb, and telomerase. This virus-mediated disruption allows genetically damaged cells to evade cell death and proliferate uncontrollably [4]. In contrast, HPV-negative (HPV−) HNSCC is primarily associated with chronic exposure to tobacco smoke and alcohol, leading to cumulative genetic and epigenetic alterations such as TP53 mutations, DNA adduct formation, aberrant methylation, and increased genomic instability compared with HPV+ tumors [5]. These biological differences contribute to the poorer prognosis and reduced therapeutic response observed in HPV-negative disease [6].

Standard treatment for HNSCC includes surgery, radiotherapy, and platinum-based chemotherapy, often combined with 5-fluorouracil. However, these approaches are frequently limited by treatment resistance and systemic toxicity, including gastrointestinal disturbances, nephrotoxicity, immunosuppression, and reduced quality of life [7–9]. For unresectable, recurrent, or metastatic disease, targeted therapies such as cetuximab and immune checkpoint inhibitors, including pembrolizumab, have been incorporated into clinical practice. Although these strategies have improved outcomes in selected patients, their clinical benefit remains limited by adverse effects, resistance, and high cost [10, 11]. Together, these limitations highlight the need for alternative or adjunct therapeutic approaches, particularly in chemoresistant HNSCC.

Cannabinoids derived from *Cannabis sativa* have attracted increasing interest due to their pharmacological properties. Among these bioactive constituents, cannabidiol (CBD) and Δ9-tetrahydrocannabinol (THC) are the most extensively studied cannabinoids. THC-based formulations are clinically used to manage chemotherapy-induced symptoms [12]. In contrast, CBD lacks psychoactive effects and presents a more favorable safety profile [13, 14].

Beyond its clinical use in symptom management, CBD has emerged as a bioactive compound with potential anticancer properties, including antiproliferative, anti-angiogenic, anti-invasive, and anti-migratory effects in multiple tumor models [15]. CBD has been shown to interact with multiple molecular targets, including cannabinoid receptor type 1 (CB1) and type 2 (CB2), transient receptor potential vanilloid (TRPV) channels, peroxisome proliferator-activated receptor gamma (PPARγ), and G protein-coupled receptor 55 (GPR55), which may influence signaling pathways associated with apoptosis and autophagy [15–17].

Despite growing evidence supporting the antitumor effects of CBD, studies specifically addressing HPV-negative and chemoresistant HNSCC remain limited [18–22]. In particular, the effects of CBD in cisplatin (CIS)-resistant HNSCC models and its impact on tumor-associated behaviors and in vivo tumor growth are not well characterized.

Therefore, the present study aimed to evaluate the effects of CBD in a CIS-resistant, HPV-negative HNSCC model (HN13) [23], focusing on tumor-associated behaviors, including migration, adhesion, metalloproteinase activity, and clonogenic survival, as well as associated cell death–related mechanisms in vitro. In addition, the antitumor activity and vascular safety of CBD were assessed using the CAM model, and the combined effect of CBD and CIS on HN13 cells was evaluated in vitro.

## Materials and methods

### Cell culture and reagents

Human HPV-negative HNSCC cell lines derived from the tongue (HN13) and hypopharynx (FaDu), as well as normal human lung fibroblasts (WI38) and keratinocytes (HaCaT), were used in this study. The HN13 and FaDu cell lines were kindly provided by Prof. Dr. Rui Manuel Reis (Barretos Cancer Hospital, Brazil). HaCaT cells were provided by Prof. Dr. Miriam Teresa Paz Lopes (Federal University of Minas Gerais), whereas WI38 cells were provided by Dr. Leandro Augusto de Oliveira Barbosa (Federal University of São João del Rei). HN13, FaDu, and WI38 cells were cultured in Dulbecco’s Modified Eagle’s Medium (DMEM, high glucose) supplemented with 10% fetal bovine serum (FBS) and 1% penicillin/streptomycin. HaCaT cells were maintained in a 1:1 mixture of DMEM and Ham’s F-12 medium supplemented with 20% FBS and 1% penicillin/streptomycin. All cell lines were cultured at 37 °C in a humidified atmosphere containing 5% CO₂ and routinely passaged at 85–95% confluence using 0.25% trypsin. CBD, CIS, dimethyl sulfoxide (DMSO), and 3-(4,5-dimethylthiazol-2-yl)-2,5-diphenyltetrazolium bromide (MTT) reagent were obtained from commercial suppliers.

### Preparation of treatments

CBD was dissolved in DMSO to prepare stock solutions and diluted in culture medium to final concentrations ranging from 1 to 50 µM, with a final DMSO concentration of 1% in all conditions. CIS was dissolved in 0.9% NaCl and used at concentrations ranging from 5 to 65 µM. Thus, 1% DMSO was used for all assays involving CBD, whereas a 0.009% NaCl vehicle control was used for the assays with CIS. For the combination assay of CIS with CBD and for the assays involving the inhibitor 3-methyladenine (3-MA) #Hy-19,312, monodansylcadaverine (MDC) #HY-D1027 staining, and spheroid and invasion assays, 1% ethanol was used as the vehicle for CBD and for the control group, due to the incompatibility of CIS with DMSO.

### Cell viability assay (MTT)

Cell viability was assessed using the MTT assay to determine half-maximal inhibitory concentrations (IC₅₀). Cells (5 × 10³ cells/well) were seeded in 96-well plates and allowed to adhere for 24 h. Cells were then treated with CBD or CIS for 24 h in medium supplemented with 0.5% FBS. Following treatment, MTT solution (0.5 mg/mL) was added for 3 h, and formazan crystals were solubilized with DMSO. Absorbance was measured at 570 nm using a microplate reader. IC₅₀ values were calculated by nonlinear regression analysis using GraphPad Prism 8.

All experiments were performed in biological triplicate with technical replicates.

### Selectivity index

The selectivity index (SI) was calculated to assess the preferential cytotoxicity of CBD toward tumor cells using the formula:

$$\mathrm{SI} = \frac{\mathrm{IC}_{50}\left(\text{non-tumor cells}\right)}{\mathrm{IC}_{50}\left(\text{tumor cells}\right)}$$

### Wound healing assay

HN13 cells (3.5 × 10⁵ cells/well) were seeded in 24-well plates and allowed to form a confluent monolayer. Linear scratches were generated using a sterile pipette tip, and cells were treated with CBD (10 µM) in medium containing 0.1% FBS to minimize proliferation. Images were acquired at 0, 24, 48, and 72 h using an inverted microscope. Wound closure was quantified using ZEN software and expressed as percentage relative to baseline.

### Gelatin zymography

Matrix metalloproteinase activity was assessed by gelatin zymography. Conditioned media were collected, and protein concentration was determined using the Bradford assay. Equal amounts of protein (20 µg) from CBD-treated samples were separated on gelatin-containing polyacrylamide gels. After electrophoresis, gels were washed to remove sodium dodecyl sulfate (SDS), incubated in activation buffer, and stained to visualize gelatinolytic activity. Bands were quantified by densitometric analysis using ImageJ. Statistical analysis is described below. Experiments were performed in biological triplicate.

### Clonogenic assay

HN13 cells (5 × 10³ cells/well) were seeded in 6-well plates and treated with CBD (10 µM) for 24 h. After treatment, cells were incubated in complete medium for 5 days to allow colony formation. Colonies were fixed, stained, photographed, and quantified using ImageJ.

### Invasion assay

Cell invasion was assessed in 24-well plates using Transwell permeable supports (Corning 3422) with an 8 μm polycarbonate membrane. The inserts were prepared with a 1:1 mixture of medium and Matrigel and incubated for 24 h. Into each insert, 1 × 10^5^ cells resuspended in DMEM containing 0% FBS and 10 µM CBD treatment were added. Culture medium containing 10% FBS was added to the plate wells as a chemoattractant. After 24 h, the inserts were washed with PBS, and the cells were fixed with 3.7% formaldehyde for 2 min and methanol for 20 min. The cells were stained with hematoxylin and eosin for 2 min. The Matrigel was carefully removed using a cotton swab, and the membranes were imaged using an EVOS microscope with a 20x objective. The stained area was measured using an automated routine developed in MATLAB and normalized to control. Data was analyzed using Prism 8.0 software.

### Hanging drop

3D spheroids were generated using the hanging-drop method. HN13 cells (0.3 × 10⁵) were resuspended in 2 mL of DMEM supplemented with 10% FBS. Droplets (20 µL) containing the cells were placed on the Petri dish lid and incubated for 72 h to allow spheroid formation. The resulting spheroids were carefully transferred to 96-well plates coated with 3% agarose to prevent adhesion. Subsequently, the spheroids were treated with 10 µM CBD for 24 h. They were imaged at 0, 24, 48, and 72 h using an inverted microscope (Axio Vert.A1) and Zen Lite 3.9 software (200× magnification; *n* = 7).

### Cell morphology and AO/PI staining

Cell morphological changes were evaluated by bright-field testing, and cell death was assessed by acridine orange/propidium iodide (AO/PI) staining. Cells were treated with CBD (10 µM) for 24 h, stained with AO/PI, and visualized by fluorescence microscopy. Cells were classified as viable, apoptotic, or necrotic based on fluorescence patterns.

### Western blotting

Protein expression was analyzed by Western blotting (WB) following 24 h treatment with CBD (10 µM). Total protein extracts were resolved by sodium dodecyl sulfate-polyacrylamide gel electrophoresis (SDS-PAGE) and transferred to nitrocellulose membranes. For the assay with the inhibitor 3-MA, cells were treated with 10 µM CBD, 3 mM 3-MA, or CBD + 3-MA for 12 h. Membranes were probed with antibodies against β-actin, Beclin-2, LC3, caspase-3, cleaved caspase-3, cleaved PARP, p53, caspase-8, cleaved caspase-8 and cyclin D1. Bands were visualized by chemiluminescence and quantified using ImageJ.

### Autophagy assessment by MDC staining

Cells were seeded in 96-well plates at a density of 5 × 10³ cells/well and incubated for 24 h. Subsequently, the cells were treated with 10 µM CBD, 3 mM 3-MA, or CBD + 3MA for 12 h. The culture medium was then removed, and 50 µM MDC, diluted in DMEM, was added. The plates were then incubated at room temperature for 15 min. Next, five images per well were captured using an inverted microscope (Axio Vert.A1) and ZEN Lite 3.9 software (200× magnification). The assay was performed in triplicate. For the MTT and WB assays, the previously described methodologies were followed using a 12-h treatment period.

### Experimental determination of synergy

The IC_50_ value of CBD was combined with each concentration of the CIS dose-response curve, whereas the IC_50_ of CIS was combined with each concentration of the CBD dose-response curve. For the combination treatment, cells were pretreated with the IC_50_ of CIS for 5 h to sensitize the cells. CIS was then removed, and CBD was added for 24 h. Subsequently, the treatment was removed, 100 µL/well of MTT was added, and the steps described above for the cell viability assay were followed to obtain the absorbance values. The data were analyzed using CompuSyn software, which generated a combination index (CI) for each drug combination. CI values < 1, = 1, and > 1 indicate synergistic, additive, and antagonistic effects, respectively.

### CAM and HET-CAM assays

The chick chorioallantoic membrane (CAM) assay was used to evaluate tumor growth, and the HET-CAM assay was employed to assess irritation scores (IS). Tumor area and vascular parameters were quantified using ZEN 3.9 and AngioTool software. Experimental procedures were conducted in accordance with institutional ethical guidelines (CEUA/UFSJ approval number 3908071123).

### Statistical analysis

All experiments were performed in at least three independent replicates. Data are expressed as mean ± standard deviation (SD). Statistical analyses were performed using GraphPad Prism 8, employing the unpaired Student’s t-test or one-way analysis of variance (ANOVA) with multiple comparisons, as appropriate. Differences were considered statistically significant at *p* < 0.05.

## Results

### CBD reduces the viability, invasion, and migration of HPV-negative HNSCC cell lines

**Fig. 1:**
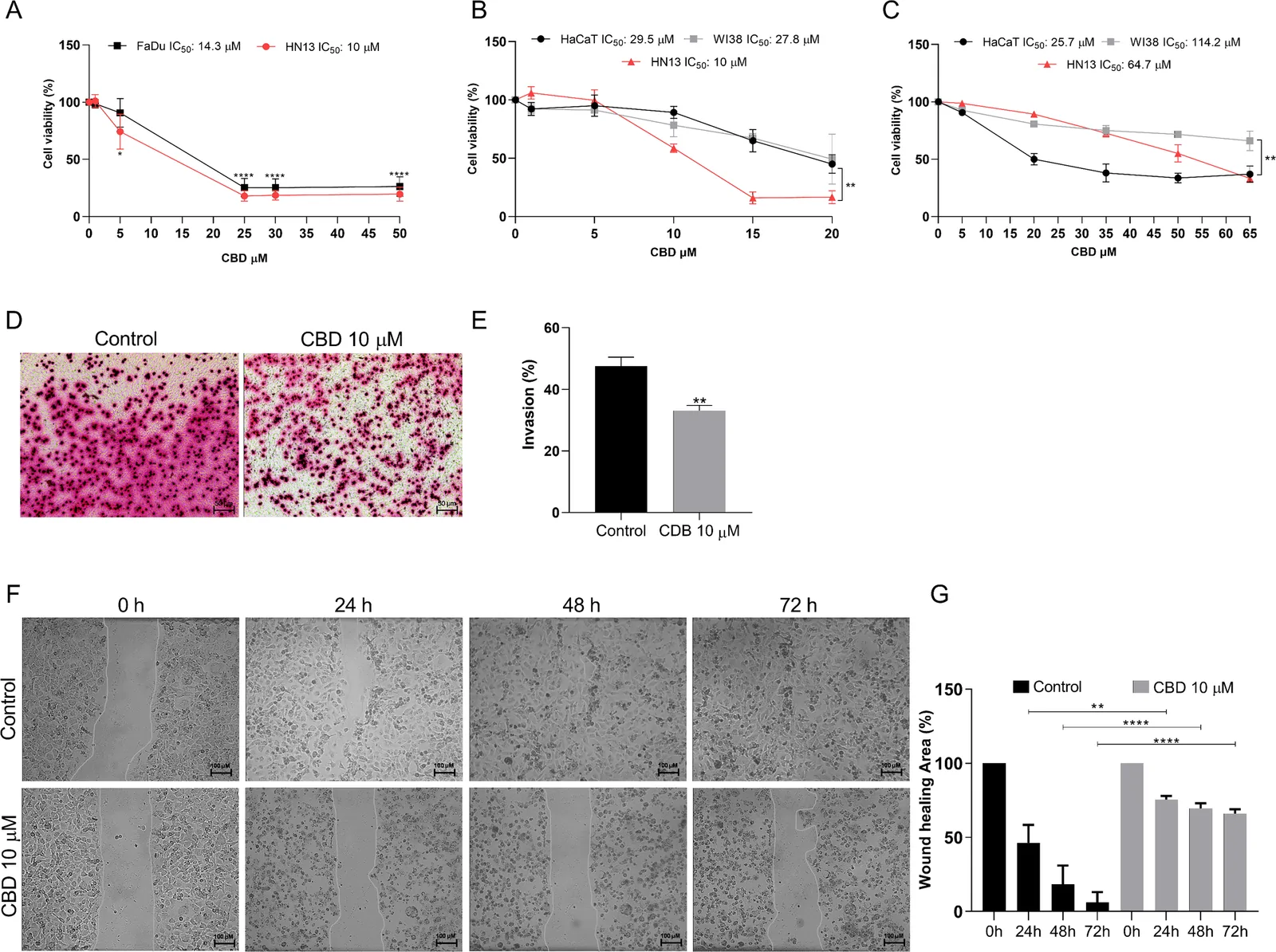
Cannabidiol selectively reduces the viability of HNSCC cells and inhibits cell invasion and migration. **A**. Dose–response curves of HN13 and FaDu cells treated with CBD (1–50 µM, 24 h); **B**. Dose–response curves of HN13, WI38, and HaCaT cells treated with CBD (1–20 µM, 24 h); **C.** Dose–response curves of HN13, WI38, and HaCaT cells treated with CIS (5–65 µM, 24 h). Cell viability was determined by MTT assay and normalized to untreated controls; **D.** Representative images of cell invasion after treatment with 10 µM CBD for 24 h; **E.** Quantification of the invaded area in pixels; **F.** Representative images of wound healing assays in HN13 cells treated with CBD (10 µM) or vehicle at 0, 24, 48, and 72 h; **G**. Quantification of wound closure expressed as percentage of remaining wound area relative to baseline. Data are presented as mean ± SD (*n* = 3 independent experiments). IC₅₀ values were calculated by nonlinear regression. Statistical analysis was performed using the unpaired t-test, and ANOVA with Tukey’s post hoc test was used for the migration assay **p < 0.01, ****p < 0.0001

Cell viability following CBD treatment was evaluated in HPV-negative HNSCC cell lines (HN13 and FaDu) using the MTT assay after 24 h exposure (1–50 µM). CBD decreased cell viability in a concentration-dependent manner, resulting in IC₅₀ values of 10 ± 1.42 µM for HN13 and 14.3 ± 2.27 µM for FaDu (Fig. 1A). Given its higher sensitivity to CBD, HN13 was selected for subsequent assays.

To assess selectivity, normal human fibroblasts (WI38) and keratinocytes (HaCaT) were treated with CBD (1–20 µM, 24 h). IC₅₀ values were 27.8 ± 4.85 µM (WI38) and 29.5 ± 3.97 µM (HaCaT), compared to 10 ± 2.59 µM in HN13 under the same conditions (Fig. 1B).

CIS cytotoxicity was evaluated as a reference (5–65 µM, 24 h). IC₅₀ values were 64.7 ± 8.31 µM for HN13, 114.2 ± 7.78 µM for WI38, and 25.7 ± 2.20 µM for HaCaT (Fig. 1C), indicating greater sensitivity of HaCaT cells to CIS compared with HN13.

### CBD shows preferential cytotoxicity toward tumor cells

Selectivity indices (SI) were calculated using IC₅₀ values. CBD showed preferential cytotoxicity toward HN13 cells relative to normal cells (SI = 2.7 for WI38/HN13 and 2.8 for HaCaT/HN13). In contrast, CIS exhibited lower selectivity (SI = 1.7 for WI38/HN13) and marked cytotoxicity toward HaCaT (SI = 0.3), consistent with its limited selectivity in epithelial cells.

### CBD inhibits the invasion and migration of HN13 cells

The effect of CBD on cell invasion and migration was evaluated using transwell and wound healing assays, respectively. After 24 h of treatment, 10 µM CBD significantly inhibited HN13 cell invasion (Fig. 1D, E, *p* = 0.0139). Furthermore, CBD-treated cells showed significantly reduced wound closure compared to controls after 24 h (*p* = 0.0019), with inhibition persisting at 48 and 72 h (Fig. 1E, F, *p* < 0.0001). After 72 h, CBD-treated cultures retained approximately 66% of the initial wound area, whereas control cells had achieved virtually complete closure.

### 3.4 CBD reduces MMP-2 and MMP-9 activity and suppresses clonogenic and 3D spheroid survival

**Fig. 2:**
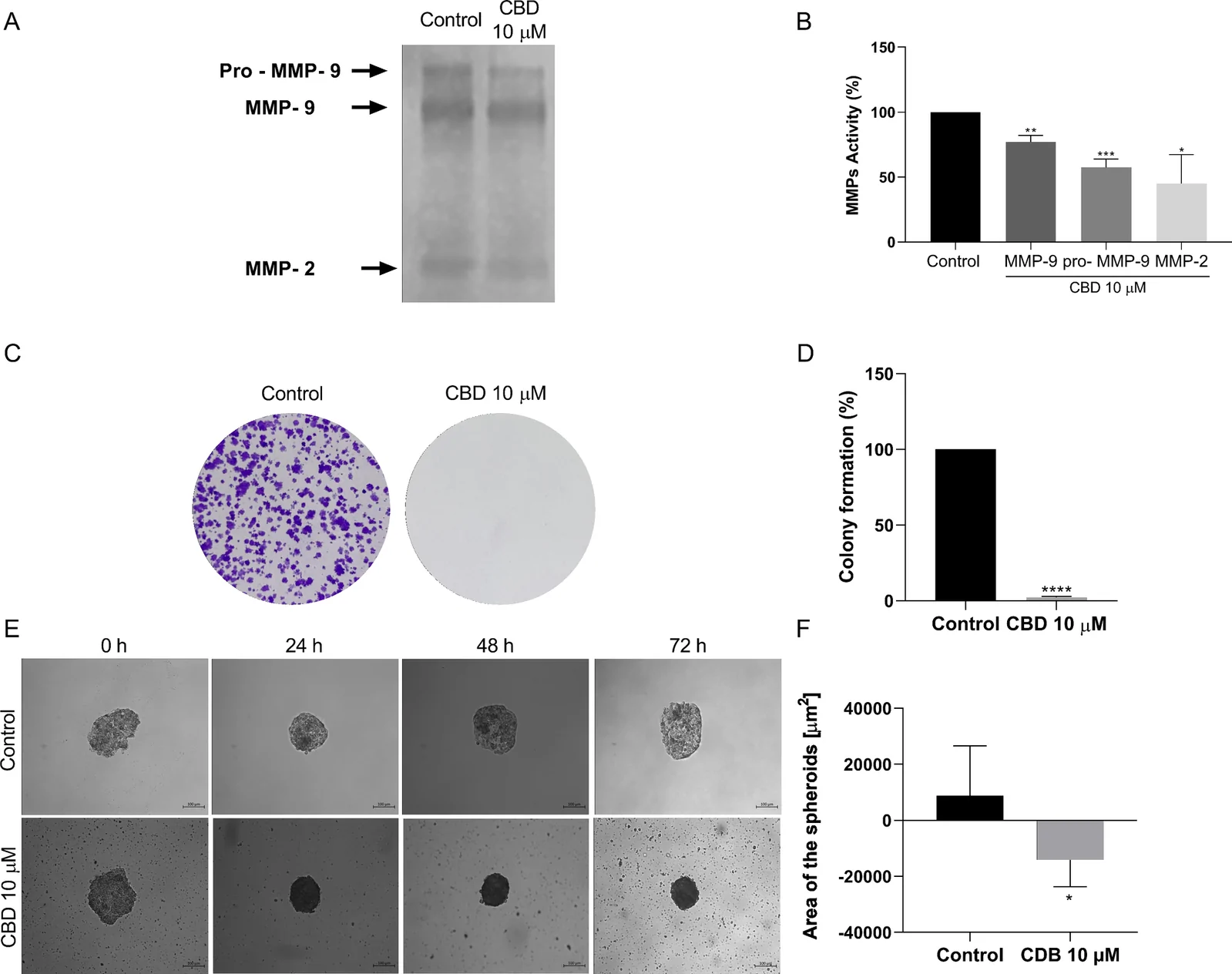
Cannabidiol reduces the gelatinolytic activity of MMP-2/MMP-9 and suppresses the clonogenic and 3D spheroid survival of HN13 cells. **(A)** Representative gelatin zymography of conditioned media from HN13 cells treated with CBD (10 µM, 24 h); **(B)** Quantification of MMP activity expressed as percentage relative to controls; **(C)** Representative images of colony formation following CBD treatment (10 µM, 24 h) and 5-day recovery; **(D)** Quantification of colony formation relative to controls; **(E)** Representative images of spheroids after 72 h of treatment with CBD; **(F)** Graph of spheroid area after 72 h of treatment; Data are presented as mean ± SD (*n* = 3 independent experiments). Statistical analysis was performed using an unpaired t-test. *p < 0.05, **p < 0.01, ***p < 0.001, ****p < 0.0001

CBD treatment (10 µM) significantly reduced gelatinolytic activity, including pro-MMP-9 (57%, *p* = 0.0003), MMP-9 (77%, *p* = 0.0013), and MMP-2 (45%, *p* = 0.0129) compared with controls (Fig. 2A, B and Supplementary 1). Furthermore, CBD treatment markedly reduced colony formation by 98% compared with controls (Fig. 2C, D, *p* < 0.0001), indicating a strong impairment of long-term proliferative capacity.

The effect of CBD on 3D spheroid cultures of HN13 cells was evaluated. While an increase in spheroid area was observed in the control group after 72 h, the treatment inhibited growth, significantly reducing the spheroid area (Fig. 2E, F, *p = 0,0131).

### CBD induces cell death–associated morphological changes

**Fig. 3:**
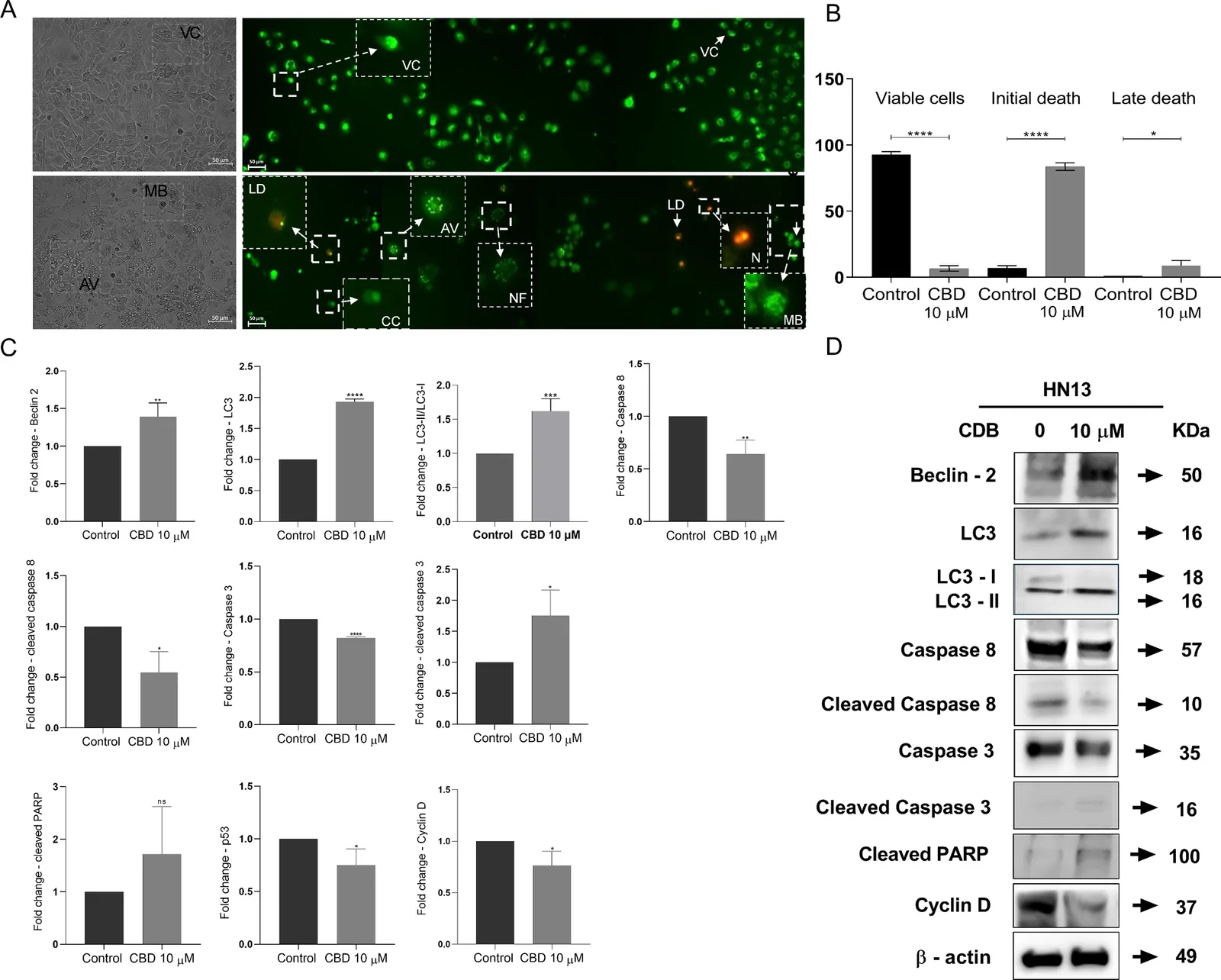
Cannabidiol modulates apoptosis, autophagy, and cell cycle–related proteins in HN13 cells. **(A)** Representative bright-field AO/PI fluorescence images of HN13 cells treated with CBD (10 µM) for 24 h compared with controls. Green indicates viable/early-stage cells (chromatin condensation (CC), membrane blebbing (MB), nuclear fragmentation (NF) and acidic vesicular structures (AV); red/orange indicates late-stage cell death (LD) and necrosis (N); **(B)** Quantification of viable, early-stage, and late-stage cell populations; **(C)** Representative Western blot images following CBD treatment (10 µM, 24 h); **(D)** Densitometric analysis normalized to β-actin and expressed relative to controls. Data are presented as mean ± SD (*n* = 3 independent experiments). Statistical analysis was performed using ANOVA with Tukey’s post hoc test for the fluorescence assay and an unpaired t-test for the Western blot, *p < 0.05, **p < 0.01, ****p < 0.000 and not significant (ns)

CBD-treated cells exhibited morphological features consistent with cell death, including cell shrinkage, chromatin condensation, membrane blebbing, and nuclear fragmentation, as well as the presence of acidic vesicular structures (Fig. 3A). Quantitative analysis showed a significant reduction in viable cells (6%, *p* < 0.0001) and an increase in early-stage (83%, *p* < 0.0001) and late-stage cell death (8%, *p* = 0.0253), as shown in (Fig. 3B).

### CBD modulates cell death and cell cycle–related proteins

Western blot analysis demonstrated that CBD treatment increased expression of LC3 (193%, *p* < 0.0001) and Beclin-2 (139%, *p* = 0.0049), consistent with modulation of autophagy-related proteins. CBD also reduced caspase-8 and cleaved caspase-8, suggesting modulation of extrinsic apoptotic signaling. In parallel, increased cleaved caspase-3 and modulation of PARP were observed. Additionally, CBD reduced expression of cyclin D1 (76%, *p* = 0.0423) and p53 (74%, *p* = 0.0484), supporting involvement of cell cycle regulation (Fig. 3C, D and Supplementary 2).

### Pharmacological inhibition of autophagy-related signaling enhances the cytotoxic and apoptotic response associated with CBD in HN13 cells

**Fig. 4:**
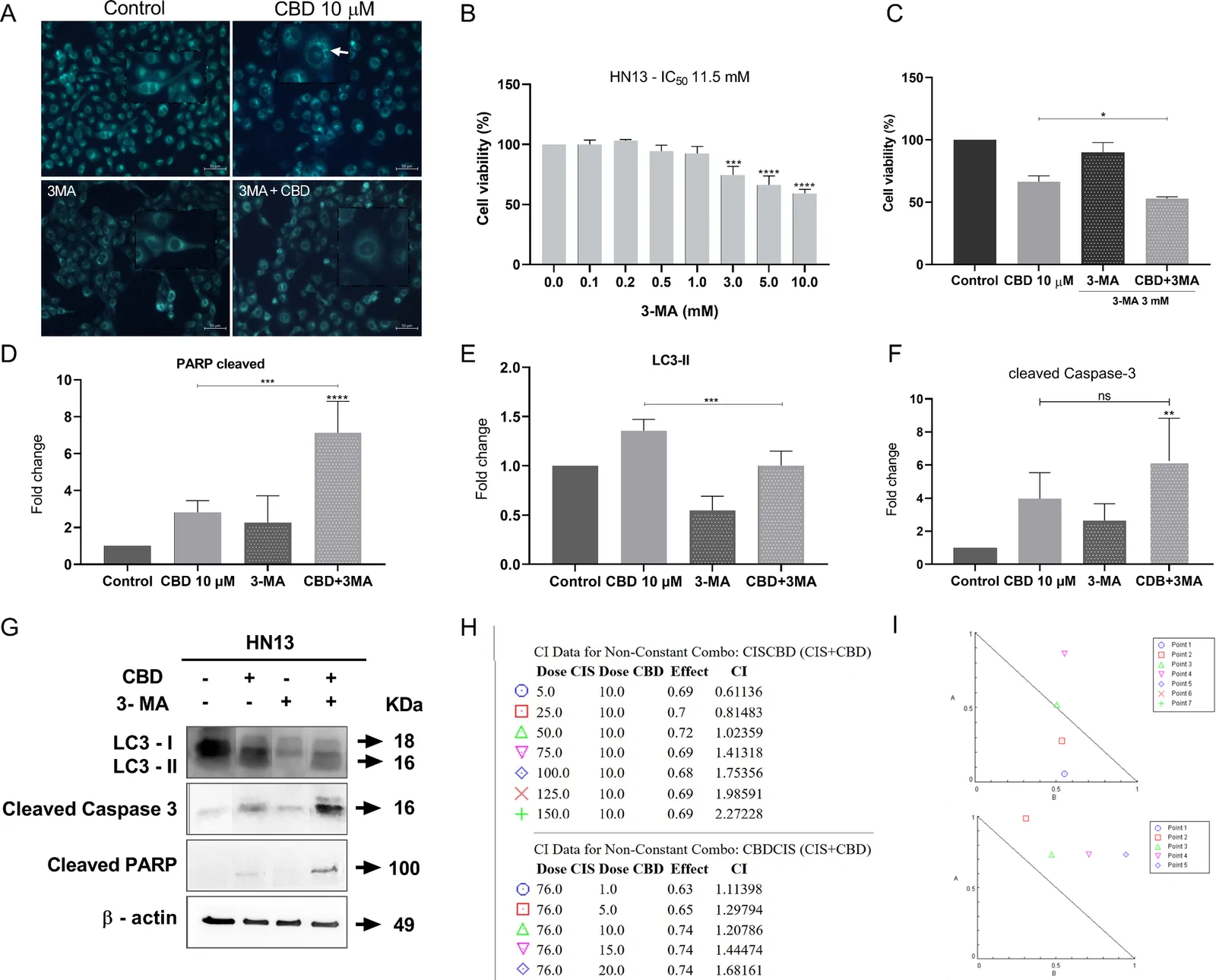
Pharmacological inhibition of autophagy-related signaling enhances the cytotoxic and apoptotic response associated with CBD, while CBD shows synergistic cytotoxic interactions with CIS in HN13 cells. **A.** Representative images from the fluorescence assay using MDC—a specific marker for autophagic vacuoles—performed after treatments with and without the inhibitor 3-MA. Arrows indicate the MDC-labeled autophagic vacuoles; **B.** Graph showing cell viability after 12 h of treatment with 3-MA at doses of 0.1, 0.2, 0.5, 1, 3, 5, and 10 mM; **C.** And treatment with CBD 10 µM, alone and in combination with the inhibitor 3-MA (3 mM), after 12 h; **D.** Graphs representing cleaved PARP expression; **E**. LC3-II; **(F)** and cleaved caspase-3 after the treatments; **(G)** Representative Western blot images following treatments; **(H)** Combinatorial effect of CIS combined with CBD, after 5 h of sensitizing treatment with CIS and 24 h with CBD. CI values generated by CompuSyn CI < 1, = 1 and > 1 indicate synergism, additive effect and antagonism, respectively; **(I)** Isobologram generated by CompuSyn, with synergistic combinations identified below the line, additive combinations on the line and antagonistic combinations above the line. Data are presented as mean ± SD (*n* = 3 independent experiments). Statistical analysis was performed using ordinary one-way ANOVA, followed by *Tukey’s multiple comparisons test*, * *p* < 0,05, **p < 0,01, ***p < 0,001 e ****p < 0,0001

We investigated whether the autophagy-related response associated with CBD treatment in HN13 cells contributed to cell death or survival. Staining with MDC (a marker of autophagic vacuoles, AV) revealed the presence of blue-fluorescent AV (indicated by the white arrow) following treatment with 10 µM CBD for 12 h (Fig. 4A). The IC_50_ value of 3-MA in HN13 tumor cells was 11.5 mM after 12 h of treatment (Fig. 4B). Consequently, we selected the lowest concentration that showed a statistically significant difference while maintaining 75% cell viability: 3 mM.

Combined treatment with CBD and 3-MA reduced autophagy-related signaling and significantly decreased cell viability compared with CBD alone (Fig. 4C, *p = 0.0280). Upon evaluating the expression of proteins involved in autophagy and apoptosis, we observed a significant reduction in LC3-II expression and an increase in PARP and caspase-3 cleavage in the group treated with CBD plus 3-MA compared with CBD alone (Fig. 4D, E, F, G). These findings suggest that autophagy-related signaling may contribute to a cytoprotective response to CBD treatment in HN13 cells, as pharmacological inhibition with 3-MA was associated with reduced LC3-II levels and enhanced apoptotic signaling.

### CBD showed a synergistic effect when combined with CIS in HN13 tumor cells

In the combination assay, the cytotoxicity of CIS was independently re-evaluated in HN13 cells using an expanded concentration range (5–150 µM). This experiment yielded an IC₅₀ of approximately 76 µM, which was used to determine the CIS concentrations for subsequent combination assays with CBD.

To assess the combined effects of CBD and CIS, two combination strategies were evaluated: the IC_50_ value of CBD was combined with each concentration of the CIS dose-response curve, whereas the IC_50_ of CIS was combined with each concentration of the CBD dose–response curve. The best synergistic effects were the combinations CISCBD1 (CI = 0.6, CIS 5 µM combined with CBD 10 µM) and CIS-CBD2 (CI = 0.8, CIS 25 µM combined with CBD 10 µM); (Fig. 4H). The software generated the CI values and the isobologram indicating the synergistic, additive and antagonistic points, represented in (Fig. 4I).

3.9 CBD reduces tumor growth and blood vessel area and does not induce vascular irritation in vivo.

**Fig. 5:**
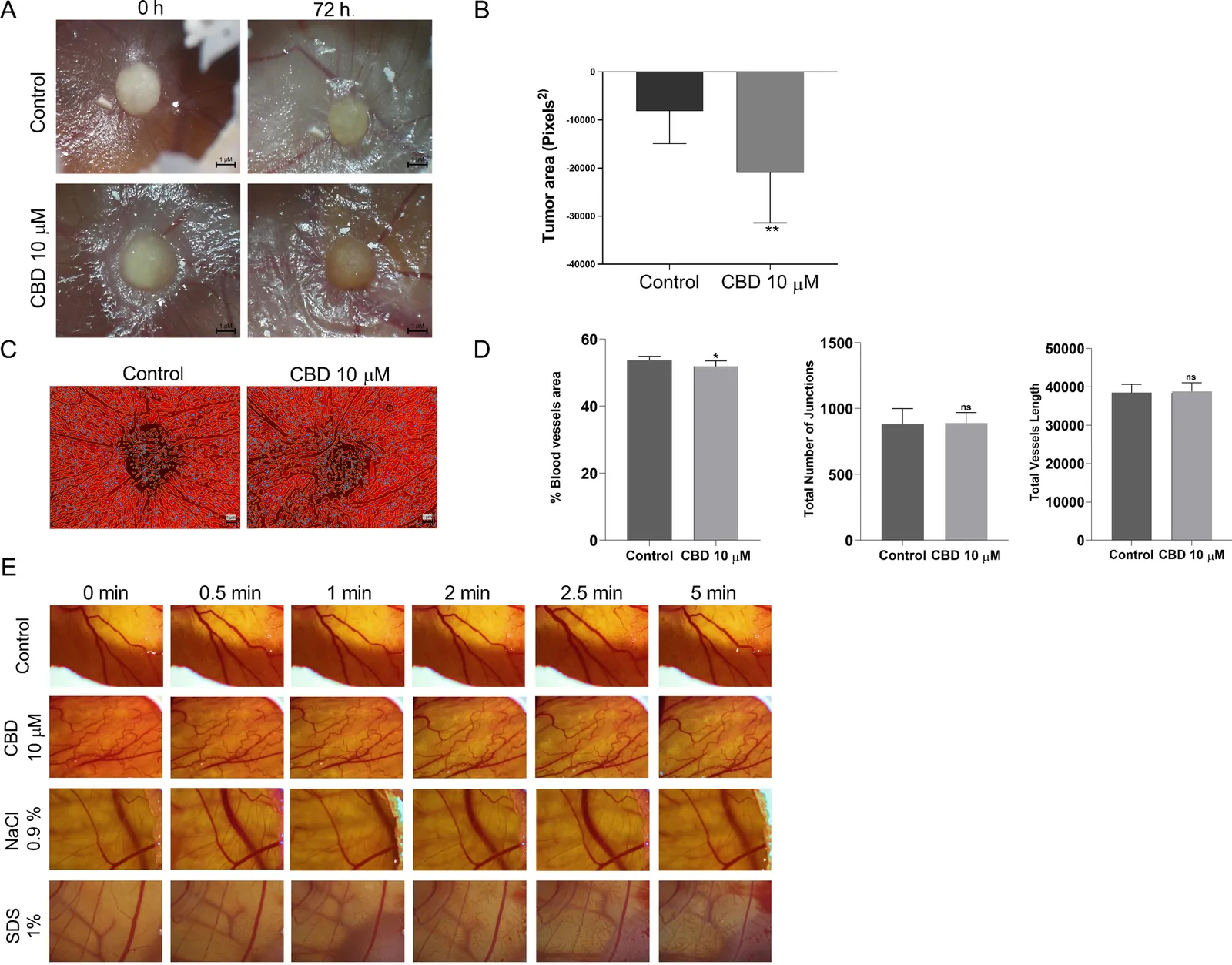
Cannabidiol reduces tumor growth and vascular area and does not induce vascular irritation in the CAM model. **(A)** Representative images of HN13-derived tumors on CAM in control and CBD-treated groups; **(B)** Quantification of tumor size after 72 h treatment (10 µM); **(C)** Representative CAM images; **(D)** Quantification of vascular area, junctions, and vessel length. Data are presented as mean ± SD (*n* = 8); **(E)** Representative images of the chorioallantoic membrane (CAM) after exposure to a control with 1% DMSO vehicle, a negative control with 0.9% NaCl, and a positive control with 1% SDS. No hemorrhage, lysis, or coagulation was observed in CBD-treated samples. Irritation scores (IS) were calculated according to standard criteria (*n* = 5). Statistical analysis was performed using an unpaired t-test. *p < 0.05, **p < 0.01; ns, not significant

In the CAM model, CBD significantly reduced tumor size compared with controls (Fig. 5B, *p* = 0.0093). CBD also reduced vascular area surrounding tumors (Fig. 5D, *p* = 0.0202), while junction number and vessel length were not significantly altered, suggesting a selective effect on vascular density (Fig. 5D, ns). Additionally, CBD did not induce hemorrhage, vascular lysis, or coagulation in the HET-CAM assay and was classified as practically non-irritating (IS = 0.57 ± 1.11), comparable to the negative and vehicle controls (IS = 0.07 ± 0). In contrast, SDS (1%) produced a strong irritant response (IS = 18.79 ± 0.40) (Fig. 5E).

## Discussion

Cannabidiol (CBD) has gained increasing attention as a biologically active compound with potential anticancer properties across multiple tumor types, including breast cancer, glioblastoma, leukemia, prostate cancer, and head and neck squamous cell carcinoma (HNSCC) [15]. Preclinical evidence suggests that CBD can modulate tumor cell viability, migration, invasion, and tumor growth in vivo, as well as influence the tumor microenvironment [24]. In this study, we demonstrated that CBD affects multiple tumor-associated processes in a CIS-resistant HNSCC model using complementary in vitro and in vivo approaches.

The pharmacokinetics and safety of CBD have been extensively investigated [25]. Perkins et al. demonstrated that an oral CBD formulation, administered in single doses of 5–20 mg/kg, was safe and well tolerated in healthy individuals. Furthermore, administration of the 20 mg/kg dose under high-fat meal conditions enhanced CBD bioavailability, resulting in a mean maximum plasma concentration of 1003 ng/mL. The CBD concentration evaluated in the present study was 10 µM, corresponding to approximately 3145 ng/mL, which is higher than the plasma concentrations reported in humans. Go et al. demonstrated that a 5 mg/kg dose of CBD inhibited tumor growth in mice xenografted with FaDu HNSCC cells [20]. Considering the translational relevance of these findings, future studies should evaluate CBD pharmacokinetics and bioavailability in mammalian HNSCC models to further assess its efficacy in vivo.

A key limitation of current HNSCC therapy is the lack of selectivity of conventional chemotherapeutics, such as CIS, which exert cytotoxic effects in both malignant and normal proliferating tissues [26]. In this study, CBD exhibited higher selectivity indices toward tumor cells compared with CIS, which showed pronounced toxicity toward normal keratinocytes. These results are in line with previous studies reporting minimal toxicity of CBD in non-tumoral epithelial and fibroblast cell models [20, 27]. Collectively, these findings suggest that CBD may offer a more favorable selectivity profile than conventional chemotherapy, although its therapeutic applicability requires further validation.

Disease recurrence and distant metastasis remain major causes of mortality in advanced HNSCC [28, 29]. Metastatic progression is a complex multistep process involving a extracellular matrix remodeling, tumor cell migration, and invasion, and clonogenic survival at secondary sites [30]. In the present study, CBD significantly inhibited HN13 cell migration, invasion, and clonogenic capacity. These findings corroborate previous studies demonstrating CBD’s ability to inhibit the migration and invasion of HNSCC cells, both HPV-positive [31] and HPV-negative [32] suggesting that CBD may modulate cellular processes associated with metastatic potential.

Importantly, these results should be interpreted as modulation of metastasis-related cellular behaviors rather than direct evidence of antimetastatic activity in vivo. Consistent with this interpretation, CBD markedly reduced the gelatinolytic activity of MMP-2 and MMP-9, enzymes that play central roles in extracellular matrix degradation, invasion, and angiogenesis [33]. Overexpression of these MMPs has been associated with aggressive behavior and poor prognosis in HNSCC [30]. Similar inhibitory effects of CBD on MMP activity and cell migration have been reported in HNSCC, breast cancer, colorectal cancer, and endothelial cell models [20, 34–36], supporting a broader role of CBD in modulating tumor progression–associated pathways. Therefore, the reduction in the motility of HN13 tumor cells, their ability to form new colonies, and MMP-2 and MMP-9 activity suggests that CBD may downregulate important cellular processes associated with metastatic progression.

3D cell culture is considered an important method for validating antitumor compounds, as it exhibits greater cell-cell interaction, hypoxic areas, and a treatment resistance profile more similar to that of tumors compared to 2D culture [37]. Previous studies have shown that tumor cells cultured in a 3D model exhibited reduced sensitivity to CBD [38] in breast tumor models, when compared to monolayer studies. Our results revealed that CBD inhibited spheroid growth within 72 h. Similarly, the same cytotoxic effects of CBD were observed in 3D breast cancer cell models [39] and of the colon [40].

Morphological analysis using AO/PI staining revealed that CBD induces cellular features consistent with programmed cell death, including chromatin condensation, membrane blebbing, nuclear fragmentation, and the presence of acidic vesicular structures [41]. These observations suggest involvement of apoptosis- and autophagy-related processes, prompting molecular investigation of key regulatory proteins.

At the molecular level, CBD treatment increased expression of LC3 and Beclin-2, indicating modulation of proteins associated with the autophagy machinery. However, as autophagic flux was not directly assessed, these findings should not be interpreted as definitive evidence of increased autophagic flux or autophagic cell death [42]. Instead, they suggest engagement of autophagy-related machinery, which may interact with apoptotic signaling. In parallel, CBD reduced expression of caspase-8 and cleaved caspase-8, which may reflect suppression of extrinsic apoptotic signaling and altered crosstalk between apoptosis and autophagy, rather than direct induction of autophagy [43].

Additionally, CBD increased cleaved caspase-3 and modulated PARP, consistent with activation of apoptotic pathways. Caspase-3 is a key executioner of apoptosis, and PARP cleavage is a hallmark of DNA damage–associated cell death [44]. Together, these findings support activation of apoptotic signaling alongside modulation of autophagy-related pathways. The modulation of apoptosis and autophagy pathways by CBD has been linked to various molecular targets, including cannabinoid receptors CB1 and CB2, vanilloid receptors (TRPV1, TRPV2, and TRPV4), as well as GPR55 and PPARγ [15]. In HNSCC, CBD (10 µM) has been shown to inhibit proliferation and cell migration by promoting apoptosis through GPR55 modulation and p38 mitogen-activated protein kinase (MAPK) signaling [45]. Thus, these molecular targets may be involved in the effects observed in the present study; however, further studies are needed to confirm their involvement in the effects of CBD in HN13 cells.

CBD additionally reduced expression of cyclin D1, a critical regulator of G1/S cell cycle progression that is frequently overexpressed in HNSCC and contributes to uncontrolled proliferation [46, 47]. Similar reductions in cyclin D1 and induction of cell cycle arrest by CBD have been reported in other HNSCC models [20]. The observed decrease in p53 expression should be interpreted with caution, as HPV-negative HNSCC commonly harbors mutant p53, and reduced protein levels may reflect altered stability or degradation rather than classical p53-dependent cell cycle control [48]. In this context, these findings suggest that the observed antitumor effects may be more closely associated with cyclin D1 modulation and caspase-3 activation than with p53-dependent signaling; however, further studies are needed.

Cancer-associated fibroblasts in the tumor microenvironment have been associated with a poorer prognosis and chemotherapy resistance in patients with HNSCC by suppressing p53- or caspase-3-mediated apoptosis [49]. Resistance to CIS is characterized by mechanisms such as DNA damage repair capacity, cell cycle checkpoint activation, and the adaptive activation of survival signaling pathways in HNSCC cells. Zhang et al. demonstrated that inhibition of checkpoint kinase 1 (CHK1) by prexasertib reversed cisplatin resistance in resistant FaDu cells [50]. Therefore, strategies capable of increasing sensitivity to chemotherapy and overcoming these resistance mechanisms are particularly relevant. Given the potential complementary cytotoxicity effects of CBD and CIS, we evaluated their combination and observed a synergistic interaction. The combination enhanced the cytotoxic effects of both CBD and CIS in HN13 cells. Thus, these findings suggest that the combination may allow for a reduction in the CIS dose while maintaining cytotoxic efficacy [51]. However, further preclinical studies are needed to determine whether lower CIS doses can maintain antitumor efficacy while reducing toxicity.

Autophagy plays a dual role in cancer, either inhibiting tumorigenesis or facilitating tumor cell survival. In this study, pharmacological inhibition with 3-MA resulted in a greater reduction in cell viability following CBD treatment, as well as increased cleavage of PARP and caspase-3. The increase in LC3-II levels and the LC3-II/LC3-I ratio, combined with the results obtained using MDC and 3-MA, supports the involvement of autophagy-related processes, although autophagic flux was not directly determined. Thus, our findings suggest that the autophagy-related response associated with CBD treatment may have a cytoprotective role in HN13 cells. Consequently, further studies using established autophagic flux assays and additional pharmacological or genetic approaches are needed to determine whether modulation of autophagy can enhance the antitumor effects of CBD [52]. However, we acknowledge as a limitation of the present study that autophagic flux was not directly assessed; the evaluation of additional markers and the use of lysosomal inhibitors and/or genetic approaches are necessary for a better understanding of autophagy in the observed processes.

The in vivo relevance of these findings was supported explored using the CAM assay, which showed that CBD significantly reduced tumor size. While mammalian models remain the gold standard for in vivo validation, the CAM model provides a rapid and cost-effective platform for early-stage evaluation [53]. Tumor angiogenesis plays an important role in tumor growth, progression, and therapeutic response across several cancer types, including HNSCC [54]. CBD treatment also reduced vascular area surrounding the tumor mass, consistent with previous reports describing anti-angiogenic effects of CBD in both in vitro and in vivo models [36, 55]. These findings suggest that CBD may influence tumor growth through both direct cellular effects and modulation of tumor-associated vasculature. However, further studies assessing specific vascular markers are needed to determine whether CBD directly inhibits tumor angiogenesis.

However, the limitations of the CAM model, particularly the absence of immune and stromal complexity, should be considered when extrapolating these results. Finally, vascular safety was assessed using the HET-CAM assay, which demonstrated that CBD did not induce hemorrhage, lysis, or coagulation and was classified as non-irritant. Given the clinical relevance of vascular toxicity and extravasation in anticancer therapies [56], these findings further support the favorable safety profile of CBD in this experimental setting.

Future studies incorporating mammalian models, three-dimensional systems, and mechanistic analyses of receptor involvement and autophagic flux are required to further define the translational potential of CBD.

## Conclusions

This study provides preclinical evidence that cannabidiol (CBD) exerts selective antitumor effects in HPV-negative HNSCC models evaluated in this study. CBD reduced tumor cell viability, impaired migration, invasion and clonogenic capacity, modulated matrix metalloproteinase activity, reduced the area of 3D spheroids and was associated with modulation of apoptosis- and autophagy-related signaling pathways in vitro. Furthermore, it acted synergistically with the chemotherapeutic agent CIS. In vivo, CBD reduced tumor growth and vascular area in the chorioallantoic membrane model without inducing vascular irritation. Collectively, these findings support CBD as a potential candidate for further investigation in HNSCC, particularly in chemoresistant settings. However, additional studies using mammalian models and three-dimensional tumor systems are required to validate the efficacy and safety of CBD, both alone and in combination with CIS, and to further clarify its mechanisms of action, receptor involvement, and translational potential.

## Supplementary Information

Below is the link to the electronic supplementary material.

Supplementary Material 1

## Data Availability

All data supporting the findings of this study are available within the paper and its Supplementary Information.The datasets generated during and/or analysed during the current study are available in the Mendeley Data repository and can be accessed during peer review via the following private link: https://data.mendeley.com/preview/7nmg2dwwsp? a=ccde40c5-28b2-4731-ad9d-5a55330231ce.Supplementary Information Polyacrylamide gels containing gelatin for the Zymogram assay were provided uncut, and Western Blot assay bands in triplicate or quadruplicate were provided in the supplementary material.

## Abbreviations

3-MA: 3-Methyladenine
ANOVA: Analysis of variance
AO/PI: Acridine orange/propidium iodide
AV: Acidic vacuoles
CAM: Chorioallantoic membrane
CB1: Cannabinoid receptor type 1
CB2: Cannabinoid receptor type 2
CBD: Cannabidiol
CC: Chromatin condensation
CI: Combination index
CIS: Cisplatin
DMEM: Dulbecco’s Modified Eagle’s Medium
DMSO: Dimethyl sulfoxide
FBS: Fetal bovine serum
GPR55: G protein-coupled receptor 55
HET-CAM: Hen’s Egg Test-Chorioallantoic Membrane
HNSCC: Head and neck squamous cell carcinoma
HPV: Human papillomavirus
IC₅₀: Half-maximal inhibitory concentration
IS: Irritation score
LC3: Microtubule-associated protein 1 A/1B-light chain 3
LD: Late-stage cell death
MAPK: Mitogen-activated protein kinase
MB: Membrane blebbing
MDC: Monodansylcadaverine
MMP: Matrix metalloproteinase
MTT: 3-(4,5-Dimethylthiazol-2-yl)-2,5-diphenyltetrazolium bromide
N: Necrosis
NF: Nuclear fragmentation
PARP: Poly(ADP-ribose) polymerase
PI: Propidium iodide
PPARγ: Peroxisome proliferator-activated receptor gamma
SD: Standard deviation
SDS: Sodium dodecyl sulfate
SDS-PAGE: Sodium dodecyl sulfate-polyacrylamide gel electrophoresis
SI: Selectivity index
THC: Δ9-Tetrahydrocannabinol
TRPV: Transient receptor potential vanilloid
WB: Western blotting
