# Supplementary material for: Cannabidiol impairs tumor-associated behaviors and modulates apoptosis- and autophagy-related pathways in cisplatin-resistant head and neck squamous cell carcinoma
Source: Med Oncol. 2026 Oct 26;43(11):360. doi: 10.1007/s12032-026-03480-7 (PMC13710429; doi:10.1007/s12032-026-03480-7)
Supplement: Supplementary file 1 [file 12032_2026_3480_MOESM1_ESM.docx]

Cannabidiol impairs tumor-associated behaviors and modulates apoptosis- and autophagy-related pathways in cisplatin-resistant head and neck squamous cell carcinoma

Edilene S. A. de Melo a (0000-0001-9786-7505), Joaquim M. D. Almeida b (0000-0001-5737-6963) and Rosy Iara M. A. Ribeiro a* (0000-0002-7374-4743)

a	Experimental Pathology Laboratory, Federal University of São João del Rei (UFSJ), Rua Sebastião Gonçalves Coelho, 400, Chanadour, Divinópolis, 35501-296, MG, Brazil.; melo.edilene97@gmail.com.

b	Plant Cell Culture Laboratory, Federal University of São João del Rei (UFSJ), Rua Sebastião Gonçalves Coelho, 400, Chanadour, Divinópolis, 35501-296, MG, Brazil.; maudall@ufsj.edu.br.

*	Correspondence: rosy@ufsj.edu.br; +55 37 99161-9155.

**Supplementary 1:** Gelatin-containing polyacrylamide uncut gels. Samples containing metalloproteinases were treated with 1% DMSO diluent in the control group in the sixth well and with 10 µM CBD in the seventh well (considering that the first and last wells of the gels were not used).

**Supplementary 2:** Repeats of western blot bands obtained on photodocument after treatment with CBD with its approximate IC_50_ of 10 µM for 24 hours.
